# Superior Interaction of Electron Beam Irradiation with Carbon Nanotubes Added Polyvinyl Alcohol Composite System

**DOI:** 10.3390/polym13244334

**Published:** 2021-12-10

**Authors:** Soo-Tueen Bee, Nicole Ooi Ker Qi, Lee Tin Sin, Hon-Meng Ng, Jun-Ven Lim, Chantara Thevy Ratnam, Chi Ma

**Affiliations:** 1 Department of Mechanical and Material Engineering, Lee Kong Chian Faculty of Engineering and Science, Universiti Tunku Abdul Rahman, Bandar Sungai Long, Kajang 43000, Selangor, Malaysia; nghongmeng@gmail.com (H.-M.N.); junvenlim@1utar.my (J.-V.L.); 2 Department of Chemical Engineering, Lee Kong Chian Faculty of Engineering and Science, Universiti Tunku Abdul Rahman, Bandar Sungai Long, Kajang 43000, Selangor, Malaysia; nicoleooi95@1utar.my; 3 Radiation Processing Technology Division Malaysian Nuclear Agency, Bangi, Kajang 43000, Selangor, Malaysia; chantara@nuclearmalaysia.gov.my; 4 School of Materials Science and Engineering, Shenyang University of Chemical Technology, Shenyang 110142, China; lg_365@163.com

**Keywords:** polyvinyl alcohol, carbon nanotubes, electron beam irradiation, nanocomposites

## Abstract

This work was conducted to investigate the effect of carbon nanotube (CNT) on the mechanical-physico properties of the electron beam irradiated polyvinyl alcohol (PVOH) blends. The increasing of CNT amount up to 1.5 part per hundred resin (phr) has gradually improved tensile strength and Young’s modulus of PVOH/CNT nanocomposites due to effective interlocking effect of CNT particles in PVOH matrix, as evident in SEM observation. However, further increments of CNT, amounting up to 2 phr, has significantly decreased the tensile strength and Young’s modulus of PVOH/CNT nanocomposits due to the CNT agglomeration at higher loading level. Irradiation was found to effectively improve the tensile strength of PVOH/CNT nanocomposites by inducing the interfacial adhesion effect between CNT particles and PVOH matrix. This was further verified by the decrement values of d-spacing of the deflection peak. The increasing of CNT amounts from 0.5 phr to 1 phr has marginally induced the wavenumber of O–H stretching, which indicates the weakening of hydrogen bonding in PVOH matrix. However, further increase in CNT amounts up to 2 phr was observed to reduce the wavenumber of O–H stretching due to poor interaction effect between CNT and PVOH matrix. Electron beam irradiation was found to induce the melting temperature of all PVOH/CNT nanocomposite by inducing the crosslinked networks.

## 1. Introduction

The Polyvinyl alcohol (PVOH) is a water soluble biodegradable synthetic polymer in which the hydroxyl groups are bonded to each monomer [1,2]. Recently, PVOH has attracted considerable attention from researchers, due to its superior performance in biocompatibility, biodegradability, hydrophilic properties, solubility in water, and being non-toxic to oral ingestion [3,4]. Based on these properties, PVOH are widely used in the application of biomedical, food packaging, etc. Moreover, the characteristics of water-soluble, hydrophilic, and biodegradable of PVOH have similar physical properties to human tissues, which have been commonly used in biomedical applications [4,5]. The usage of PVOH in biomedical applications, such as wound dressing, artificial organs, and drug delivery systems have been widely developed [6]. However, the application of PVOH has been restricted for further advance application due to its relatively poor mechanical properties, such as tensile strength, flexibility, modulus, etc. [1]. Numerous studies have been conducted in order to improve the mechanical properties of PVOH by adding reinforcing fillers such as montmorillonite, carbon nanotubes, etc. [6,7,8,9].

Currently, several approaches, such as the addition of reinforcing fillers and the application of high energy irradiation technique, were used by numerous researchers to induce the mechanical properties of polymer matrix [10,11,12,13]. The application of carbon nanotubes, as reinforcing fillers in polymer composites, to induce the mechanical properties such as tensile strength, Young’s modulus, impact strength, hardness, etc., has been intensively investigated by numerous researchers [14,15,16]. According to previous studies [16,17,18], the incorporation of carbon nanotubes has effectively improved the mechanical properties of various types of polymer composites by enhancing the interfacial bonding among the carbon nanotubes particles and polymer matrix. As reported by Sui et al. [18], they found that the carbon nanotubes could stiffen and strengthen the polymer matrix by applying the concept of micromechanics. Besides, the embedding of carbon nanotubes particles in a polymer matrix was also found to drastically increase the mechanical properties by inducing the interfacial friction and shear force between the particles and polymer matrix [18]. According to Li et al. [19], the addition of carbon nanotubes could delay the propagation of cracking effects on the reinforcement polymer matrix due to the good interfacial interaction effect between the carbon nanotubes particles and polymer matrix. Another research conducted by Sugiura et al. [20] also mentioned that the formation of three-dimensional cellular structures by carbon nanotubes in natural rubber matrix was found to effectively improve the mechanical strength of natural rubber. The three-dimensional cellular structures of carbon nanotubes could evenly transfer the applied load from the particles to the matrix.

Besides, the high energy irradiation, such as electron beam, gamma ray, or X-ray is widely applied on polymer composite for modifying the mechanical properties in order to expand their applications for more advance technology [12,21,22,23]. Electron beam irradiation is a form of ionization energy, which characterizes with low penetration and high dosage rates. According to previous studies conducted by various researchers [1,12,13], the application of electron beam irradiation has significantly improved the mechanical and thermal properties of polymer composites by forming the three-dimensional networks. The formation of three-dimensional networks could improve the mechanical and thermal properties of polymer composites, as indicated by Bee et al. (2014). According to Bee et al. [1], the formation of crosslinking networks has significantly induced the mechanical properties of PVOH-MMT blends by promoting the intercalation effect of MMT particles in a polymer matrix. However, the application of higher electron beam irradiation dosages on PVOH-MMT blends has exhibited an inferior effect on mechanical properties due to the occurrence of chain scissioning reaction [1,24]. In this work, the incorporation of carbon nanotubes was used as the primary enhancement approach to improve the mechanical, physical, and thermal properties of PVOH. This is because the mechanical properties of PVOH depend on the structural arrangement of the polymer matrix [1]. The addition of carbon nanotubes into PVOH matrix is expected to enhance PVOH matrix with higher tensile strength, rigidity, and stiffness due to its large surface areas, high mechanical interlocking effect in polymer matrix, and chemical stability with local stiffening effect in polymer matrix [25,26]. Thus, it has been widely employed and studied in the application of polymer composites. However, there is no study conducted and focused on the electron beam irradiation with incorporated carbon nanotubes nanocomposites on the mechanical-physico properties and thermal properties of the PVOH composites. Therefore, this study was carried out to investigate the effect of the carbon nanotubes and electron beam irradiation on the mechanical and physical properties of PVOH composites.

## 2. Experimental

### 2.1. Materials

In this research work, fully hydrolysed polyvinyl alcohol (PVOH) resin in powder form with Sekisui Selvol^TM^ 325 was selected as the polymer base. This grade of PVOH resin was manufactured by Sekisui Specialty Chemicals America LLC, Houston, TX, United States. Multi-walled carbon nanotubes (CNTs) with particles diameter of outer diameter: >50 nm, inner diameter: 5–15 nm, length: 10–20µm, special surface area: >40 m^2^/g produced using chemical vapour deposition method was purchased from XFNANO Materials Tech Co., Ltd., China. CNTs was used as sole reinforcing filler in this research work.

### 2.2. Preparation of PVOH/CNTs Composites

The PVOH/CNTs composites were prepared with a constant amount of 100 part per hundred resin (phr) PVOH resin and various amounts of CNTs (0.5, 1.0, 1.5, 2.0 phr) via solution cast method. The mixture of PVOH/CNTs solution were prepared by dissolving the PVOH resin in powder form into hot distilled water using a water bath at the temperature of 97 ± 2 °C. The mixture of PVOH solution was then stirred with a stirrer with driving motor at a rotation speed of 800 rpm to ensure all the PVOH resin powder has fully dissolved into hot distilled water. Subsequently, the CNTs powder was added into the PVOH solution and the mixture PVOH/CNTs solution was further stirred and heated in water bath at temperature of 97 ± 2 °C for 30 min until all the CNTs powder was dispersed in the PVOH solution. After that, the dispersed mixture of PVOH/CNTs solution was cast into film form by using a Teflon mould. The cast PVOH/CNTs solution on the Teflon mould was then dried in an oven at the temperature of 50 °C for 24 h. The dried sample was put into the sealed plastic bags and stored in a desiccator cabinet at the room temperature, at 25 °C, to control the humidity of the dried samples.

### 2.3. Characterization Testing

#### 2.3.1. Tensile Test

The tensile properties of the cast samples were conducted in accordance to the standard of ASTM D882 by using Instron Tensile Microtester, Norwood, MA, USA with model of 5848. Before the tensile testing, the thickness and width of specimen was measured using measuring instrument. During the tensile testing, the operating crosshead speed was set at a constant speed of 50 mm/min. The tensile testing was conducted under the cell load of 5 kN. The tensile strength and Young’s modulus of each sample was taken as an average of 5 specimens.

#### 2.3.2. X-ray Diffraction (XRD) Test

X-ray diffraction analysis (XRD) test was performed to determine the dispersion state of carbon nanotubes in the sample using Shimadzu XRD 6000 X-ray Diffractometer (XRD), Kyoto, Japan. The XRD spectra of all PVOH/CNTs composites were recorded with the diffractometer using Cu-Kα radiation generator with wavelength of 1.542 Å. The scanning rate was set at constant rate of 1.2°·min^−1^ and the scattering angles (2θ) range was fixed at the range of 0–40°. The operating current and acceleration voltage of the Cu-Kα radiation generator were set at 30 mA and 40 kV, respectively. The d-spacing, d of crystallites was calculated using the Bragg’s equation, as shown in Equation (1). While, the inter-chain separation, R of crystallites was determined by using Klug and Alexander equation, as shown in Equation (2).

(1) $$d=\frac{\lambda }{2\cdot \sin\theta }$$

(2) $$R=\frac{5\cdot \lambda }{8\cdot \sin\theta }$$

where λ is 1.542 Å and θ is the Bragg angle in radians.

#### 2.3.3. Differential Scanning Calorimetry (DSC) Test

The thermal properties of the PVOH/CNTs samples were investigated using the Mettler Toledo brand differential scanning calorimeter with model of DSC823. The samples of PVOH/CNTs composites were initially weighted onto a standard aluminium pans. A sealed empty pan was used as a reference pan for the DSC test. Before the scanning process, the nitrogen gas was purged into the combustion column at the constant rate of 20 mL/min. The DSC examination was carried out at the temperature range of 25 to 240 °C at scanning rate of 20 °C/min.

#### 2.3.4. Fourier Transform Infrared Spectroscopy (FTIR) Analysis

Fourier transform infrared (FTIR) analysis was performed to investigate the existence of specific chemical groups in the samples of all PVOH/CNTs composites by using Nicolet FTIR Spectrometer. The specimen of PVOH/CNTs composites was placed at the centre of the ATR plate. The samples of PVOH/CNTs composites were scanned under the band region of 4000 to 400 cm^−1^.

#### 2.3.5. Scanning Electron Microscopy Analysis (SEM)

The surface morphologies of the samples of PVOH/CNTs composites were observed using a Hitachi scanning electron microscope with a model of S3400. Firstly, the fractured surfaces of PVOH/CNTs composites were cut into smaller portion using sample cutter. The cut samples of PVOH/CNTs composites were then mounted onto the specimen stub with the fractured surface is facing up by using carbon tape. The specimen stubs with mounted samples were then coated with a layer of gold and palladium using EMITECH SC7620 Sputter Coater (Quorum Technologies, East Sussex, UK) and the coated samples was ready for SEM scanning. The coated samples of PVOH/CNTs composites were then scanned with electron beam voltage of 30 kV. The SEM micrographs were then recorded at the magnification of 3000 times.

## 3. Results and Discussion

### 3.1. Tensile Properties

#### 3.1.1. Tensile Strength

**Figure 1:**
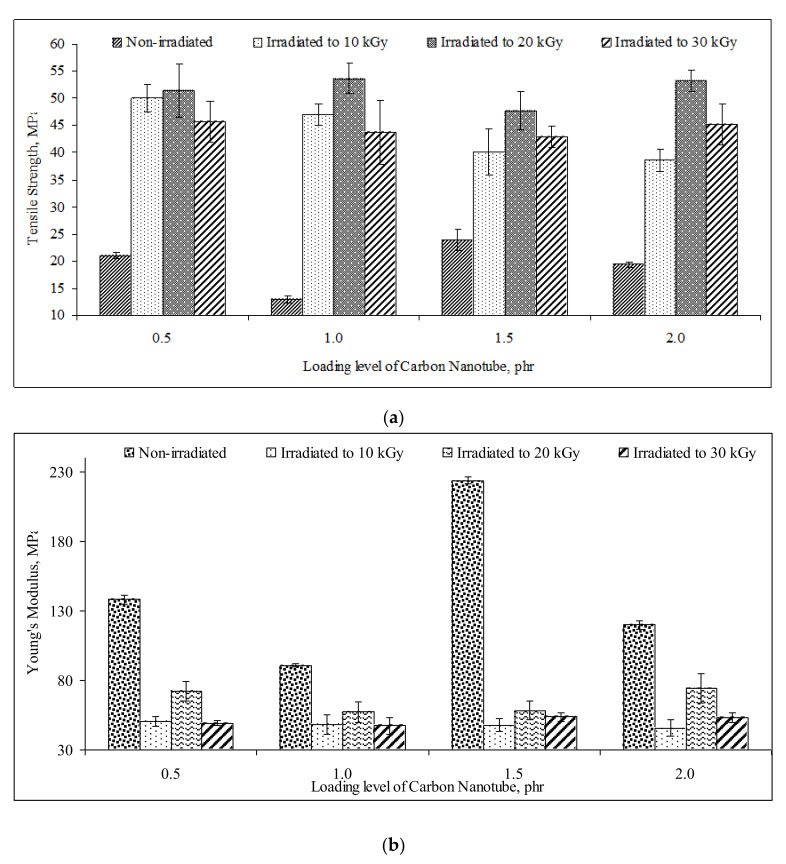
Effects of increasing electron beam irradiation dosages on (**a**) tensile strength and (**b**) Young’s modulus of polyvinyl alcohol (PVOH) nanocomposites added with various loading levels of carbon nanotubes (CNTs).

By referring to Figure 1a, the increasing of carbon nanotubes loading level from 0.5 phr to 1 phr has gradually decreased the tensile strength of all PVOH/CNTs composites. The incorporation of 1 phr carbon nanotubes particles into PVOH matrix has showed a significant decrement and reached the lowest tensile strength. This indicates that the addition of 1 phr carbon nanotubes particles did not effectively promote interfacial adhesion with PVOH matrix, resulting in the applied straining stress being unable to transfer evenly throughout the polymer matrix [1,27]. However, further increasing the loading level of carbon nanotubes from 1 phr to 1.5 phr has gradually increased the tensile strength of non-irradiated PVOH/CNTs composites, as shown in Figure 1a. This is due to the presence of a higher amount of carbon nanotubes particles in PVOH matrix has efficiently provided the bridging effect in PVOH matrix and induced the transferring of straining stress from PVOH matrix to carbon nanotubes particles within the PVOH/CNTs composites [28] Furthermore, the higher amounts of dispersed carbon nanotubes in PVOH matrix could also contribute to a stronger mechanical interlocking interaction effect with polymer matrix by providing a better reinforcement effect to PVOH matrix (Zhao et al., 2017). By referring to Figure 1a again, the tensile strength of non-irradiated PVOH/CNTs composites was marginally decreased when the loading level of carbon nanotubes increased from 1.5 phr to 2 phr. This is mainly attributed to high amounts of carbon nanotubes particles in PVOH matrix could also significantly reduce the interfacial interaction of carbon nanotubes particles and PVOH matrix. This is mainly caused by the carbon nanotubes particles tend to agglomerate together into larger agglomerates particles. The agglomeration of carbon nanotubes particles in PVOH matrix has resulted in lowering the specific area of carbon nanotubes particles and thus weakened the Van der Waals binding in polymer matrix. Moreover, the irregular shape and size of carbon nanotubes aggregates can also reduce the efficiency of straining stress to be transferred from carbon nanotubes to matrix. As a result, the reinforcing effect of carbon nanotubes particles in PVOH matrix has also significantly decreased the tensile strength of PVOH/CNTs composites. [14,29].

By observing the Figure 1a, the tensile strength of all PVOH/CNTs composites was observed to dramatically increase with increasing of electron beam irradiation dosages up to 20 kGy. This indicates that the application of electron beam irradiation technique could tremendously enhance the tensile strength of all PVOH/CNTs composites by introducing the formation of crosslinking network in PVOH matrix. The electrons released by the electron beam accelerator could attack the hydroxyl groups (–OH) on the PVOH macromolecular chains to generate the polymeric free radicals. These generated polymeric free radicals would react together and form a crosslinked bonding between the PVOH macromolecular chains [1,7]. The formation of crosslinked networks in a PVOH matrix could tremendously improve the interfacial interaction between the carbon nanotubes particles and PVOH matrix. The enhancement in interfacial adhesion effect between the carbon nanotubes particles and PVOH matrix could effectively transfer the applied straining stress from carbon nanotubes particles to polymer matrix and thus increased the tensile strength of all PVOH/CNTs composites. However, the tensile strength of all PVOH/CNTs composites was found to significantly decrease when further irradiated from 20 kGy to 30 kGy, as shown in Figure 1a. This is attributed to the available amounts of –OH groups in the PVOH matrix to generate polymeric free radicals that were significantly reduced at higher irradiation dosages [1,23]. This was caused by the excess electrons released from electron beam accelerator tended to attack and breakdown the backbone chains of PVOH macromolecules to generate the polymeric free radicals. The breakdown of PVOH backbone chains could reduce the molecular sizes of PVOH macromolecular chains and further weakened the intermolecular bonding between PVOH chains. Subsequently, the efficiency in transferring of the applied straining stress throughout the whole PVOH matrix evenly has also been significantly reduced [21,22].

#### 3.1.2. Young’s Modulus

By referring to Figure 1b, all non-irradiated PVOH/CNTs composites was observed to pose the highest value in Young’s modulus when compared to the all irradiated PVOH/CNTs composites. This indicates that the application of electron bean irradiation on polymer matrix of all PVOH/CNTs composites has dramatically reduced the rigidity of PVOH/CNTs composites. This might be due to the presence of crosslinked PVOH chains in the polymer matrix of all irradiated PVOH/CNTs composites, which could further extend the slippage effect of polymer chains when subjected to straining [21] and thus significantly reduce the stiffness of all irradiated PVOH/CNTs composites. As observed in Figure 1b, the application of electron beam irradiation dosage of 10 kGy has drastically decreased the Young’s modulus of all PVOH/CNTs composites. This indicates that the formation of low degrees of crosslinking networks in PVOH matrix unable to provide efficient restriction effect to the mobility of PVOH macromolecular chains during straining. Furthermore, the presence of a lower degree of crosslinking networks could also promote the slipping effect of PVOH macromolecular chains when subjected to straining, as discussed earlier, and thus, decrease the modulus (or stiffness) of all PVOH/CNTs composites. However, further increasing the electron beam irradiation dosages from 10 kGy to 20 kGy has gradually increased the Young’s modulus of all PVOH/CNTs composites as illustrated in Figure 1b. This is attributable to higher degree of crosslinking networks induced by irradiation dosage of 20 kGy could marginally decrease the extendibility in the slippage effect of PVOH macromolecular chains of polymer matrix by slightly restricting the PVOH chains mobility when subjected to straining. This has further increased the modulus and stiffness of PVOH/CNTs composites as higher straining stress is required to strain the polymer matrix. From the Figure 1b, the Young’s modulus of PVOH/CNTs composites was found to significantly decrease again with further increasing of irradiation dosage from 20 kGy to 30 kGy. This is because the chains scissioning reaction is more pre-dominated than crosslinking reaction when subjected to higher irradiation dosages (≥30 kGy) due to the limited available amounts of O–H groups in PVOH matrix [1,23]. The limited availability of O–H group amounts in the PVOH matrix caused the excess released electron to attack the backbone chains of PVOH to generate the shorter polymeric free radicals and subsequently reduced the molecular size of PVOH chains. This could further reduce the restriction effect on the movement of PVOH macromolecular chains during straining and thus significantly decrease the rigidity of all PVOH/CNTs composites at higher irradiation dosage of 30 kGy. Furthermore, the lower molecular size of PVOH macromolecular chains could also reduce the Van der Waals binding in the PVOH matrix and thus weaken the stiffness of all PVOH/CNTs composites.

On the other hand, it can be obviously found that the incorporation of 1.5 phr carbon nanotubes particles has highly increased the Young’s modulus of non-irradiated PVOH/CNTs composites, as shown in Figure 1b. This indicates that the addition of a higher loading level of carbon nanotubes up to 1.5 phr could effectively form an inter-blocking network of carbon nanotubes in PVOH matrix. The existence of an effective inter-blocking network in polymer matrix could effectively restrict the movements of PVOH macromolecular chains during straining and indicate the high rigidity behaviour of PVOH/CNTs composites [30]. However, the Young’s modulus of non-irradiated PVOH/CNT composites was gradually decreased when the loading level of carbon nanotubes was further increased from 1.5 phr to 2 phr, as shown in Figure 1b. This might be due to the high loading level of carbon nanotubes (≥2 phr) in PVOH matrix, which could significantly induce the agglomeration of carbon nanotubes particles in PVOH matrix and thus, weaken the interfacial adhesion effect between the carbon nanotubes particles and PVOH matrix. This could further reduce the inter-blocking effect of carbon nanotubes in PVOH matrix and cause the applied load to be unable to efficiently transfer from PVOH matrix to carbon nanotubes particles in PVOH matrix [30]. By observing the Figure 1b, the increasing of carbon nanotubes particles loading level was found to provide insignificant effect in enhancing the Young’s modulus of all irradiated PVOH/CNTs composites. This might be attributed to the formation of crosslinking networks in the PVOH matrix and could induce the interfacial interaction between the carbon nanotubes particles and PVOH matrix by strongly tightening the carbon nanotubes particles in PVOH matrix [19]. This could cause the polymer matrix to be unable to stop the crack growth by prolonging the tensile strain and thus, reduce the rigidity or modulus of all irradiated PVOH/CNTs composites.

### 3.2. XRD Analysis

**Figure 2:**
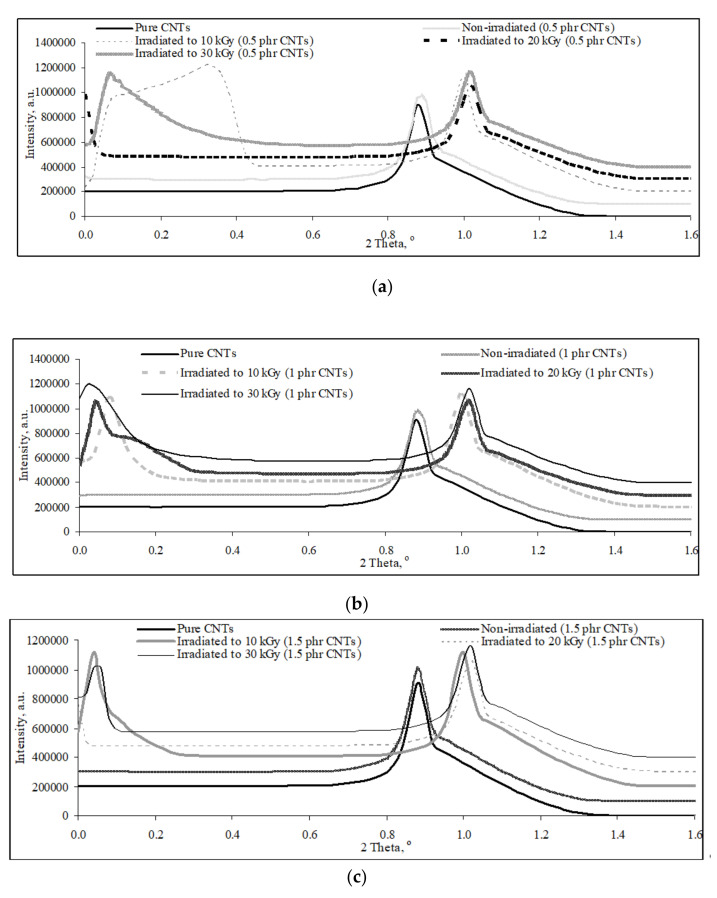

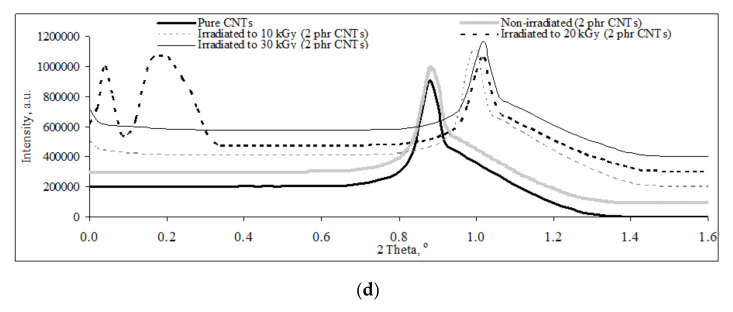
Effects of increasing loading level of carbon nanotubes (CNTs) on XRD curves for non-irradiated and irradiated polyvinyl alcohol (PVOH) nanocomposites. (**a**) 0.5 phr CNTs; (**b**) 1.0 phr CNTs; (**c**) 1.5 phr CNTs; (**d**) 2.0 phr CNTs.

By referring to the Figure 2, a deflection peak was observed to appear at 2θ range of 0.89^o^ to 1.05°on the XRD curve of pristine carbon nanotubes and all PVOH/CNTs composites. The 2θ, d-spacing, interchains separation of pristine carbon nanotubes, and all non-irradiated and irradiated PVOH/CNTs composites were tabulated in Table 1. As observed in Table 1, the increasing of carbon nanotubes particles from 0.5 phr to 1 phr has slightly increased the d-spacing and interchain separation of deflection peak of non-irradiated PVOH/CNTs composites, which indicates the good dispersion effect of carbon nanotubes particles in PVOH matrix. However, the d-spacing and interchains separation of deflection peak for non-irradiated PVOH/CNTs composites were marginally decreased when the loading level of carbon nanotubes increased from 1 phr to 2 phr. The reduction in d-spacing and interchain separation of deflection indicates the poor interaction effect between carbon nanotubes particles and PVOH matrix due to the agglomeration of carbon nanotubes in PVOH matrix. The agglomeration of carbon nanotubes particles in PVOH matrix could reduce the interlayer spacing and gap distance between that of carbon nanotubes particles in PVOH matrix [1,28].

On the other hand, the increasing of electron beam irradiation up to 30 kGy was observed to significantly shift the deflection peak of all PVOH/CNTs composites to higher 2θ value as shown in Figure 2. This finding could also be further evident by the calculated values of d-spacing and inter-chain separation for the deflection peak in Figure 2, as tabulated in Table 1. The d-spacing and inter-chain separation of deflection peak of all PVOH/CNTs composites were gradually decreased when irradiated up to 30 kGy as shown in Table 1. This indicates the application of electron beam irradiation could reduce the gap distance between the carbon nanotubes particles in PVOH matrix. This is attributed to the formation of crosslinking networks induced by electron beam irradiation could tighten the carbon nanotubes particles dispersed in PVOH matrix. In other words, the formation of crosslinking networks could further promote the interfacial interaction effect between the PVOH matrix and carbon nanotubes [1]. However, the d-spacing and interchain separation of the deflection peak on XRD curve of 0.5 phr carbon nanotubes added PVOH/CNTs composites were slightly increased when further irradiated from 20 to 30 kGy. This might be attributable to the pre-dominant of chains scissioning reaction of PVOH macromolecular chains when subjected to higher irradiation dosages (≥30 kGy), which could weaken the tightening effect of carbon nanotubes particles in PVOH matrix by crosslinked chains [1]. Thus, the gap distance of carbon nanotubes was slightly increased at higher irradiation dosages (≥30 kGy).

**Figure 3:**
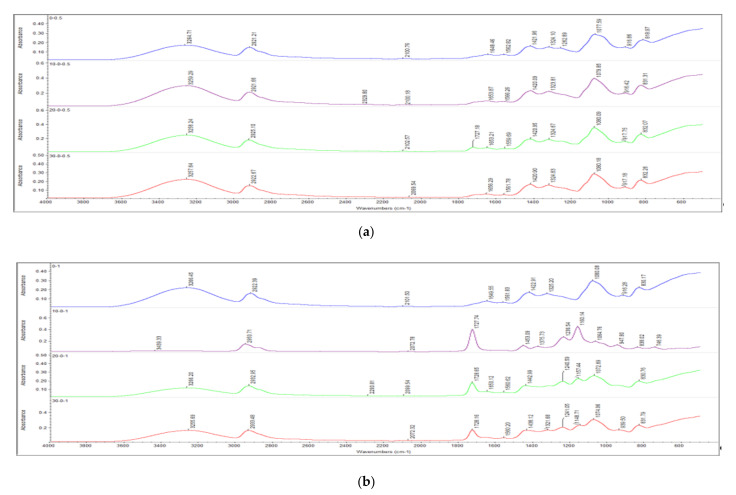
Effects of increasing electron beam irradiation dosages on FTIR spectrum of (**a**) 0.5 phr CNTs and (**b**) 1.0 phr CNTs added polyvinyl alcohol (PVOH) nanocomposites.

**Figure 4:**
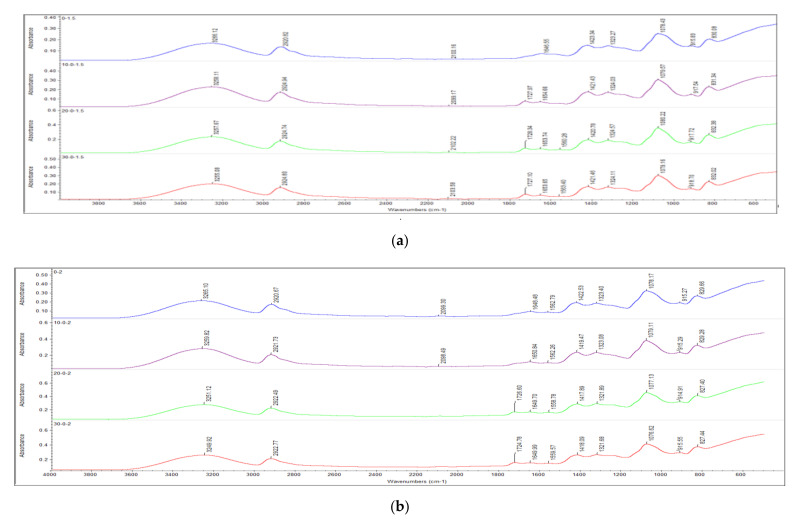
Effects of increasing electron beam irradiation dosages on FTIR spectrum of (**a**) 1.5 phr CNTs and (**b**) 2.0 phr CNTs added polyvinyl alcohol (PVOH) nanocomposites.

Figure 3 and Figure 4 illustrate the infrared spectrum of all non-irradiated and irradiated PVOH/CNTs composites when added with the increasing loading level of carbon nanotubes. As shown in Figure 3 and Figure 4, a prominent peak can be observed as occurring on the FTIR spectrum of all the samples of PVOH/CNTs composites at band region of 3000 cm^−1^ to 3600 cm^−1^, which is attributed to the stretching of hydroxyl group (O–H) [31,32]. The addition of carbon nanotubes particles into polymer matrix of PVOH was observed to change the bonding strength of O–H stretching within the lattice structure, as shown in Figure 3 and Figure 4. The effect of the carbon nanotubes loading level on hydroxyl group (O-H) stretching band of all non-irradiated and irradiated PVOH/CNTs composites was summarized in Table 2. The increasing of carbon nanotubes from 0.5 to 1 phr has marginally increased the wavenumber of the O–H stretching of non-irradiated PVOH/CNTs composites from 3264.71 to 3266.45 cm^−1^. The increasing of the wavenumber of O–H stretching indicates the weakening effect of hydroxyl groups (O–H) in the polymer matrix. This shows that the incorporation of higher carbon nanotubes particles (≥1 phr) in PVOH matrix could slightly reduce the occurrence of hydrogen bonding in PVOH matrix. This is due to the presence of higher amounts of carbon nanotubes in the PVOH matrix could disturb the interaction between the PVOH macromolecular chains in polymer matrix and thus weaken the hydrogen bonding in PVOH matrix. As the loading level of carbon nanotubes was further increased from 1 to 2 phr, the wavenumber of hydroxyl group (O-H) stretching was slightly decreased as tabulated in Table 2. This might be due to the carbon nanotubes particles in polymer matrix that tended to agglomerate into larger agglomerates particles when added with higher loading level (>1 phr). The agglomeration of carbon nanotubes particles in PVOH matrix has weakened the interfacial adhesion effect between carbon nanotubes particles and PVOH matrix and thus, the disturbance effect of carbon nanotubes in PVOH matrix. Subsequently, the hydrogen bonding in PVOH was slightly increased.

The wavenumber of hydroxyl group (O–H) stretching of all PVOH/CNTs composites was observed with general trend to gradually decrease when electron beam irradiated up to 30 kGy, as illustrated in Figure 3 and Figure 4 and Table 2. The decrement in wavenumber of the hydroxyl group (O–H) stretching indicates the presence of stronger hydrogen bonds inside PVOH matrix [33]. This might be due to the application of the electron beam irradiation technique on polymer matrix could induce the formation of crosslinking networks in polymer matrix by generating the polymeric free radicals. The generated polymeric free radicals could react together by forming a “linkage bridge” to connect the PVOH macromolecular chains together, and this has further decreased the gap distance between the PVOH macromolecular chains. Subsequently, the shorter gap distance between the PVOH chains could further promote the presence of hydrogen bonding in PVOH matrix and thus reduce the hydroxyl group (O–H) stretching in polymer matrix. Besides, the peak absorbance of hydroxyl group (O–H) stretching was observed to gradually decrease when subjected to increasing irradiation dosage up to 30 kGy, as depicted in Figure 3 and Figure 4. This observation also indicates that the available amounts of hydroxyl (O–H) groups in PVOH matrix were significantly reduced when irradiated to higher irradiation dosages. This is because the released electrons would attack the hydroxyl functional group (O–H) in polymer matrix of PVOH to generate the polymeric free radicals. These polymeric free radicals would react together by forming a crosslinked network in PVOH matrix and subsequently reduce the available amounts of hydroxyl group (O–H) in PVOH matrix.

On the other hand, the presence of C–H bonding peak can also be clearly seen at the band region of 2900 cm^−1^ on FTIR spectrums, as depicted in Figure 3 and Figure 4. By referring to Table 2, the increasing of carbon nanotubes loading level from 0.5 to 1 phr has slightly increased the wavenumber of C–H bonds stretching for non-irradiated PVOH/CNTs composites. This indicates the good interaction effect between the carbon nanotubes particles and PVOH macromolecular chains inside polymer matrix. This is due to how the carbon nanotubes particles, with non-polar nature, could interact well with the non-polar saturated C–H bonds of PVOH macromolecular chains and thus, induce the strength of C–H stretching of non-irradiated PVOH/CNTs composites [3]. When the loading level of carbon nanotubes was further increased from 1 to 2 phr, the wavenumber of C–H bonds in polymer matrix of non-irradiated PVOH/CNTs composites was marginally reduced from 2922.39 to 2920.67 cm^−1^, as observed in Table 2 This showed that the incorporation of higher loading level of carbon nanotubes into PVOH matrix of non-irradiated PVOH/CNTs composites has slightly weakened the interaction effect between carbon nanotubes particles by agglomerating the carbon nanotubes particles into larger particles. The agglomeration of carbon nanotubes particles in PVOH matrix has weakened the interaction of carbon nanotubes particles and PVOH matrix by changing the intermolecular vibrations within the PVOH matrix. Thus, the changes in intermolecular vibrations in the polymer matrix have further weakened the original intermolecular interaction in the PVOH matrix and induced the wavenumber of C–H stretching.

On the other side, the wavenumber of C–H stretching of all PVOH/CNTs composites was observed to significantly increase when an electron beam irradiated up to 30 kGy except 0.5 phr carbon nanotubes added PVOH/CNTs composite, as observed in Figure 3 and Figure 4 and Table 2. The increasing of C–H stretching in polymer matrix indicates the improvement on the interaction effect between carbon nanotubes particles and PVOH chains in the polymer matrix of all PVOH/CNTs composites. This is attributable to the formation of crosslinking networks in PVOH matrix induced by electron beam irradiation could effectively induce the interaction effect between the carbon nanotubes and thus induce the bonding strength of C–H stretching. However, the C–H stretching bonds of lower amount of carbon nanotubes (0.5 phr) added PVOH/CNTs composites was marginally decreased when an electron beam further irradiated from 20 to 30 kGy. This indicates that the application of higher irradiation dosages (≥30 kGy) could slightly weaken the interaction effect between the carbon nanotubes and PVOH macromolecular chains in polymer matrix of low carbon nanotubes added PVOH/CNTs composites. When subjected to higher irradiation dosages, the availability of O–H groups in PVOH matrix was significantly limited and caused the excess electrons that tended to attack the backbones chains of PVOH chains to generate polymeric free radicals and thus reduced the degree of crosslinking networks in PVOH matrix. Subsequently, the reduction in the degree of crosslinking in PVOH matrix could slightly weaken the interaction effect between the carbon nanotubes particles and PVOH chains and thus reduce the bonding strength of C–H stretching.

### 3.3. Scanning Electron Microscopy (SEM) Observation

**Figure 5:**
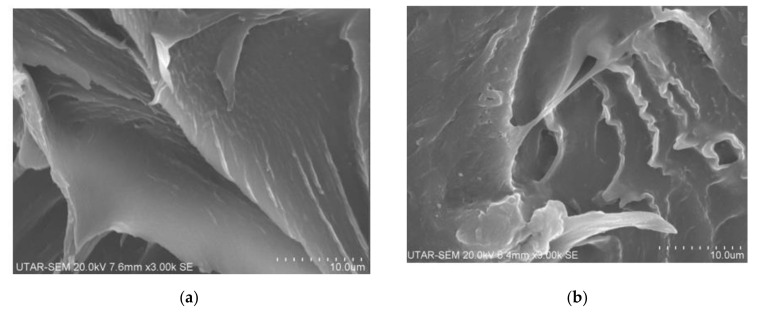

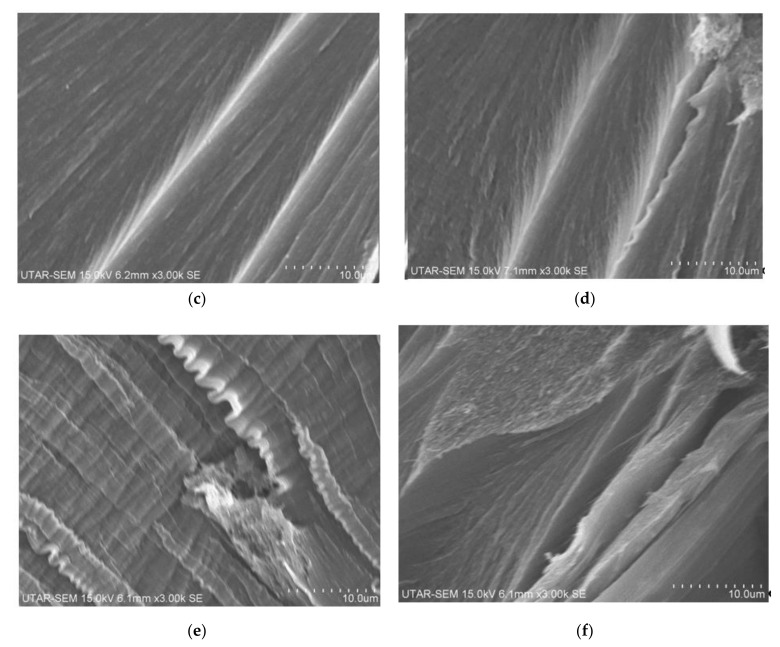
SEM micrographs of non-irradiated PVOH/CNT nanocomposites added with (**a**) 0.5 phr CNT, (**b**) 2.0 phr CNT, 10 kGy irradiated PVOH/CNT nanocomposites added with (**c**) 0.5 phr CNT, (**d**) 2.0 phr CNT, 30 kGy irradiated PVOH/CNT nanocomposites added with (**e**) 0.5 phr CNT and (**f**) 2.0 phr CNT.

Figure 5 illustrates the fractured surface morphologies of PVOH/CNTs composites added with 0.5 and 2 phr (low and high) loading levels of carbon nanotubes when irradiated to irradiation dosages of 10 and 30 kGy. By referring to Figure 5a,b, the tearing effect can be clearly observed to occur on the fractured surface of 0.5 and 2 phr carbon nanotubes added PVOH/CNTs composites. The presence of tearing effect is mainly attributed to the elongation ability of polymer matrix of non-irradiated PVOH/CNTs composites under straining. However, the tearing effect on the fractured surface of 0.5 phr carbon nanotubes that added PVOH/CNTs composites was observed to be larger, more continuous, and less in amount when compared to the 2 phr carbon nanotubes added PVOH/CNTs composites. From Figure 5a, the smooth fractured surface of non-irradiated PVOH/CNTs composites, added with lower carbon nanotubes particles, indicates the good dispersion effect of carbon nanotubes particles in PVOH matrix at lower loading level of carbon nanotubes. At a higher amount of carbon nanotubes particles, the carbon nanotubes particles tended to agglomerate together into larger carbon nanotubes agglomerates particles as observed in Figure 5b. The agglomeration of carbon nanotube particles in the PVOH matrix has significantly weakened the interfacial adhesion effect between carbon nanotubes particles and PVOH matrix, and it has acted as stress concentration points in the polymer matrix during straining [11]. This further caused the applied straining stress unable to be evenly transferred from polymer matrix to agglomerates particles and tear the PVOH matrix that interfaced with the agglomerates particles of carbon nanotubes. Thus, the PVOH/CNTs composites, added with higher carbon nanotubes (2 phr), was found to pose less continuous and smaller tearing effect than the 0.5 phr carbon nanotubes added PVOH/CNTs composites. On the other hand, the tearing effect on the all PVOH/CNTs composites (0.5 and 2 phr) was found to disappear when subjected to electron beam irradiation dosage up to 10 kGy. Besides, the fractured surface of 10 kGy irradiated samples were also observed to be significantly more continuous and smoother than all non-irradiated samples. This indicates that the formation of crosslinking networks, induced by a low irradiation dosage of 10 kGy, has significantly extended the elongation ability of PVOH/CNTs composites by delaying the tearing effect of polymer matrix. However, the fractured surface of all PVOH/CNTs composites (0.5 and 2 phr) was found to breakdown without a significant elongation effect during straining. This observation indicates the application of electron beam irradiation dosage up to 30 kGy has severely degraded and embrittled the polymer matrix of all PVOH/CNTs composites.

### 3.4. Differential Scanning Calorimetry

**Figure 6:**
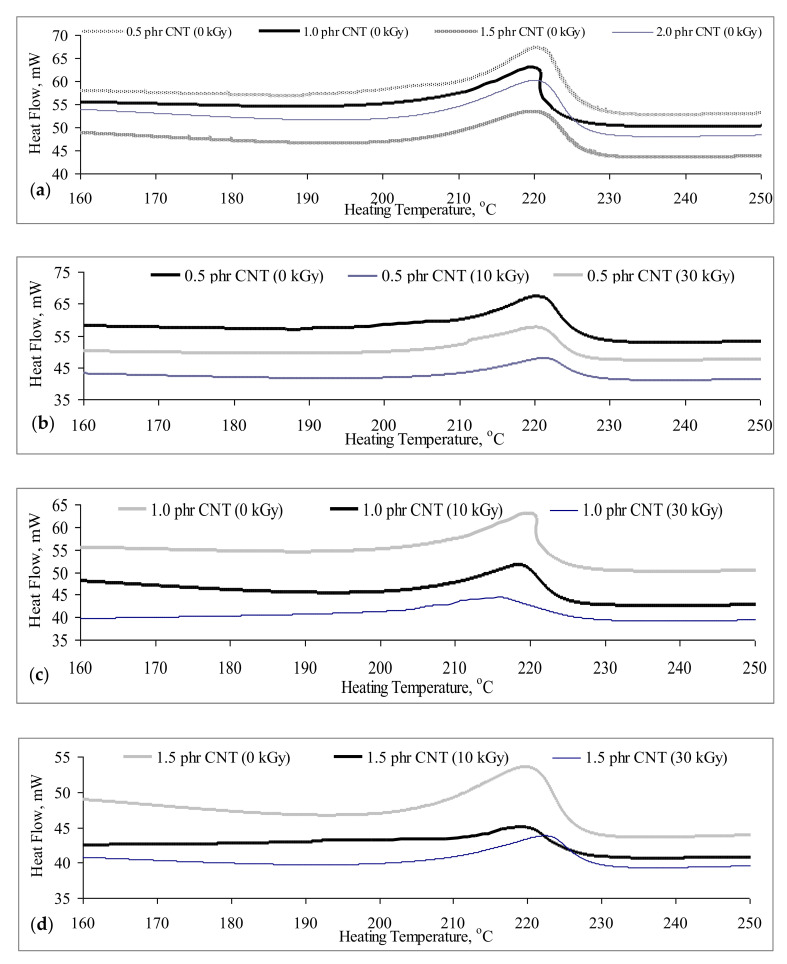

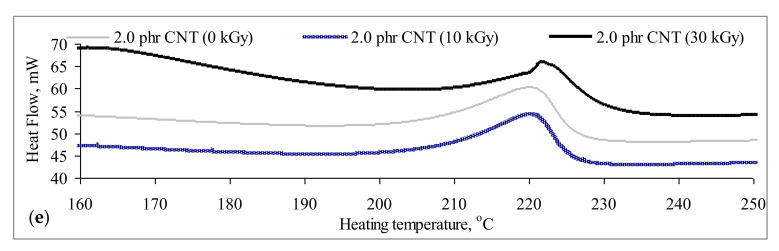
(**a**–**e**): DSC thermograms of carbon nanotubes (CNT) added PVOH blends under various irradiation dosages.

From Figure 6, a significant endothermic peak can be observed to occur on the DSC thermograms of all carbon nanotubes (CNT) added PVOH nanocomposites. The presence of this endothermic peak also indicates the melting state of all PVOH/CNT nanocomposites occurred at the temperature range from 210 to 230 °C. By observing the Figure 6a, the endothermic peak of all PVOH/CNT nanocomposites were significantly broadened when the loading level of CNT increased from 0.5 to 1.5 phr. This indicates that the increasing CNT amount from 0.5 to 1.5 phr has significantly disturbed the presence of hydrogen bonding in polymer matrix of PVOH/CNT nanocomposites by broadening the endothermic peak. This is attributed to the presence of CNT particles could reduce the hydrogen bonding in PVOH matrix by introducing the interaction bonding between the CNT particles and PVOH matrix. This can be further proved that the enthalpy of melting of non-irradiated PVOH/CNT nanocomposites was gradually increased with an increasing CNT amount, as tabulated in Table 3. This also indicates that the addition of CNT has significantly induced the required thermal energy to provide sufficient amount of kinetic energy by discharging the polymer molecules from the ordered crystallite structure [24]. Moreover, the melting endothermic peak with broader area might also attribute to enhancement of the arrangement crystallite structure with higher molecular weight. This indicates that the higher of CNT particles could effectively interact with PVOH chains by increasing the intermolecular bonding in the polymer matrix of PVOH nanocomposites [33]. As results, higher thermal energy is required to overcome the intermolecular bonding between CNT particles and PVOH matrix. The melting temperature of non-irradiated PVOH/CNT nanocomposites was also found to slightly increase with increasing of CNT loading level, as shown in Table 3. However, the enthalpy of melting of non-irradiated PVOH/CNT nanocomposites was observed to marginally decrease when the loading level of CNT particles increased from 1.5 to 2 phr. This might be due to the agglomeration of CNT particles at higher loading level could disrupt and hinder the ordered chains arrangement structure in PVOH matrix [24]. Thus, the required thermal energy to overcome the intermolecular bonding of 2 phr CNT added PVOH nanocomposites has been significantly reduced.

By referring to Figure 6b, the intensity and area of endothermic peak of 0.5 phr CNT added PVOH/CNT nanocomposites was observed to significantly reduced and broadened when irradiated to 10 kGy. This indicates that the introduction of low degree of crosslinking network in PVOH matrix has significantly reduced the required thermal energy to overcome the internal bonding (intermolecular and hydrogen) in polymer matrix PVOH/CNT nanocomposites. However, the melting peak was observed to significantly shift from 211.5 to 222.9 °C when the 0.5 phr CNT added PVOH/CNT nanocomposites were further irradiated from 10 to 30 kGy. This might be due to higher irradiation dosage up to 30 kGy has highly promoted the formation of crosslinking networks in PVOH matrix by releasing more electron to form polymeric free radicals [22]. A higher degree of crosslinking networks formed in PVOH matrix could induce the intermolecular interaction between PVOH molecules and CNT particles in polymer matrix. Thus, higher thermal energy is needed to breakdown the intermolecular interaction in polymer matrix of 30 kGy irradiated 0.5 phr CNT added PVOH/CNT nanocomposites. On the other hand, the increasing of electron beam irradiation dosage, up to 30 kGy, was observed to gradually decrease the melting enthalpy of PVOH/CNT nanocomposites added 1 and 1.5 phr CNT, as tabulated in Table 3. This is mainly due to the good dispersion of CNT particles, which significantly reduced the presence of hydrogen bonding of PVOH/CNT nanocomposites. At higher irradiation dosages, more electrons that were released by the electron beam accelerator tended to attack O–H groups in PVOH matrix to generate the polymeric free radicals. This has further reduced the presence of hydrogen bonding in polymer matrix of PVOH/CNT nanocomposites and thus reduced the required thermal energy to overcome the hydrogen bonding in PVOH matrix [3]. This can be further proved by the significant reduction in intensity and area of endothermic peak, as shown in Figure 6c,d. By referring to Figure 6e, the application of electron beam irradiation dosages of 10 and 30 kGy has significantly increased the enthalpy of melting and melting temperature of 2 phr CNT added PVOH/CNT nanocomposites. This also indicates that the formation of crosslinking networks in higher CNT amounts (2 phr) added PVOH/CNT nanocomposites could intermolecularly induce the interaction between PVOH matrix and CNT particles by promoting better interaction bonds in the PVOH/CNT system. Thus, more thermal energy is required to excite of polymer molecules by breaking these formed interaction bonds [3]. Therefore, it can be concluded that the thermal stability of the PVOH/CNT nanocomposites can be significantly increased with the increasing loading level of CNT loading level and application of suitable electron beam irradiation dosage.

## 4. Conclusions

In this study, the effect of incorporating the carbon nanotubes particles and electron beam irradiation on the physical-mechanico and thermal properties of PVOH/CNTs composites have been intensively investigated. The tensile strength and Young’s modulus of non-irradiated PVOH/CNTs composites were gradually increased with increasing of carbon nanotubes loading level up to 1.5 phr. This is due to the higher loading level of carbon nanotubes particles (15 phr) could induce the effective amounts of interlocking carbon nanotubes particles in PVOH matrix by efficiently transferring the applied straining stress from particles to PVOH matrix as evident in SEM analysis. However, the tensile strength and Young’s modulus of non-irradiated PVOH/CNTs composites were gradually decreased when the loading level of carbon nanotubes increased from 1.5 phr to 2 phr due to the agglomeration of carbon nanotubes at higher loading level of carbon nanotubes (as evident in SEM observation). The agglomerated carbon nanotubes could act as stress concentration points in PVOH matrix during straining and thus decrease the efficiency of transferring the straining stress evenly throughout polymer matrix of PVOH/CNTs composites. Besides, the introduction of electron beam irradiation has tremendously improved the tensile strength of all PVOH/CNTs composites by improving the interfacial interaction between carbon nanotubes particles and PVOH matrix as evident in XRD analysis with the decrement of d-spacing and interchains separation of detected crystallite. This is due to the formation of crosslinking networks could tighten the carbon nanotubes particles in PVOH matrix and subsequently induce the interfacial adhesion effect between the particles and PVOH matrix. However, further increments in irradiation dosage from 20 kGy to 30 kGy has gradually decreased the tensile strength of all PVOH/CNTs composites due to pre-dominant of the chains scissioning reaction over the crosslinking process in PVOH matrix at higher irradiation dosage. By referring to FTIR analysis, the hydroxyl group (O-H) stretching band of PVOH/CNTs composites has significantly increased when the amounts of carbon nanotubes particles increased from 0.5 phr to 1 phr, which indicates the weakening effect of the hydrogen bonding in the PVOH matrix. This is due to the presence of carbon nanotubes could hinder the hydrogen bonding between the PVOH macromolecular chains and thus reduce the hydrogen bonding within PVOH matrix. However, a higher amount of carbon nanotubes particles in PVOH matrix has slightly decreased the hydroxyl (O–H) stretching of all PVOH/CNTs composites, due to the agglomeration of carbon nanotubes particles in PVOH matrix. This is because the agglomeration of carbon nanotubes particles has weakened the interaction effect of carbon nanotubes particles and PVOH macromolecular chains in PVOH matrix and thus slightly induced the presence of hydrogen bonding in the PVOH matrix. The application of electron beam irradiation has shown a significant increment in the melting temperature of all PVOH/CNT nanocomposites. This indicates that the formation of crosslinking networks in polymer matrix could promote the intermolecular interaction effect, between CNT and PVOH matrix, and thus, effectively induces the melting temperature of PVOH/CNT nanocomposites. Besides, the increasing of CNT loading level from 0.5 to 1.5 phr has gradually induced the enthalpy of melting of non-irradiated PVOH/CNT nanocomposites. This is due to the presence of higher CNT amounts, with higher crystallite structure in PVOH matrix, which could promote intermolecular bonds in PVOH matrix of non-irradiated PVOH/CNT nanocomposites. Subsequently, higher thermal energy is needed to rupture the intermolecular bonding between CNT particles and PVOH matrix.

## Figures and Tables

**Table 1:** Effects of increasing loading level of carbon nanotubes (CNTs) on the 2 theta (2θ), *d*-spacing, and interchain separation of deflection peak (002) on XRD curves for non-irradiated and irradiated polyvinyl alcohol (PVOH) nanocomposites, as depicted in Figure 2.

Samples/Polyvinyl Alcohol Nanocomposites		2 Theta (2θ), ^o^	*d*-Spacing, Å	Inter-Chain Separation (*R*), Å
Loading Level of CNTs*, phr*	Electron Beam Irradiation Dosage, kGy			
Pure Carbon Nanotubes (CNTs)		0.9112	96.873	121.04
0.5	0	0.8990	98.188	122.69
	10	1.0281	85.858	107.28
	20	1.0369	85.130	106.37
	30	1.0245	86.160	107.66
1.0	0	0.8918	98.980	123.68
	10	1.0263	86.009	107.47
	20	1.039	84.958	106.16
	30	1.0469	84.317	105.35
1.5	0	0.8984	98.253	122.77
	10	1.0255	86.076	107.55
	20	1.0344	85.336	106.63
	30	1.0369	85.130	106.37
2.0	0	0.9018	97.883	122.31
	10	1.0300	85.675	107.08
	20	1.0438	84.567	105.67
	30	1.0288	85.800	107.21

Remarks: CNTs*: Carbon nanotubes; phr*: parts per hundred resin.

**Table 2:** Wavenumber of O–H stretching and C–H stretching of all carbon nanotubes added polyvinyl alcohol (PVOH) nanocomposites when subjected to various electron beam irradiation dosages.

Loading Level of Carbon Nanotubes (CNTs), phr	Electron Beam Irradiation Dosage, kGy	Wavenumber, cm^−1^	
		O–H Stretching	C–H Stretching
0.5	0	3264.71	2921.21
	10	3259.29	2921.66
	20	3258.24	2925.10
	30	3257.64	2922.67
1.0	0	3266.45	2922.39
	10	3257.91	2923.93
	20	3256.20	2932.95
	30	3255.69	2933.48
1.5	0	3266.12	2920.82
	10	3258.11	2924.94
	20	3257.67	2924.74
	30	3255.68	2924.80
2.0	0	3265.10	2920.67
	10	3259.82	2921.73
	20	3251.12	2922.49
	30	3249.92	2922.17

**Table 3:** Effects of increasing loading level of carbon nanotubes (CNTs) on melting temperature and enthalpy of melting for non-irradiated and irradiated polyvinyl alcohol (PVOH) nanocomposites.

Samples/Polyvinyl Alcohol Nanocomposites		Melting Temperature, °C	Enthalpy of Melting, J/g
Loading Level of CNTs*, phr*	Electron Beam Irradiation Dosage, kGy		
0.5	0	221.50	21.69
	10	221.51	14.07
	30	222.92	35.83
1.0	0	220.5	22.38
	10	219.75	17.70
	30	217.98	13.60
1.5	0	221.00	29.46
	10	221.26	15.76
	30	223.79	12.78
2.0	0	221.00	26.59
	10	221.06	40.33
	30	222.12	31.80

Remarks: CNTs*: Carbon nanotubes; phr*: parts per hundred resin.
